# LINC00221 silencing prevents the progression of hepatocellular carcinoma through let-7a-5p-targeted inhibition of MMP11

**DOI:** 10.1186/s12935-021-01819-w

**Published:** 2021-04-09

**Authors:** Lin Yang, Hailong Si, Meng Ma, Yu Fang, Yina Jiang, Jintao Wang, Cheng Zhang, Haijuan Xiao

**Affiliations:** 1 Department of Hepatobiliary Surgery, Shaanxi Province, Xianyang Central Hospital, No. 78, Renmin East Road, Weicheng District, Xianyang, 712000 People’s Republic of China; 2 Department of Oncology, Shaanxi Province, Affiliated Hospital of the Shaanxi University of Traditional Chinese Medicine, No. 2, Weiyang West Road, Xianyang, 712000 People’s Republic of China; 3 Diagnostic Teaching and Research Unit, Shaanxi University of Traditional Chinese Medicine, Xianyang, 712046 People’s Republic of China

**Keywords:** Hepatocellular carcinoma, Long noncoding RNA LINC00221, Let-7a-5p, Matrix metalloproteinase-11, Cell cycle, Cell migration

## Abstract

**Background:**

Microarray profiles of hepatocellular carcinoma (HCC) identified that long intergenic noncoding RNA 00221 (LINC00221) was upregulated. Herein, we aimed to identify the functional significance and underlying mechanisms of LINC00221 in HCC.

**Methods and results:**

Human HCC samples had increased expression of LINC00221. Effects of LINC00221 on HCC cellular functions were analyzed using gain- and loss-function approaches. LINC00221 knockdown repressed HCC cell growth, migration, and invasion and enhanced their apoptosis. This anti-tumor effect was validated in vivo. Online prediction showed the potential binding relationship between LINC00221 and let-7a-5p, as well as that between let-7a-5p and matrix metalloproteinase 11 (MMP11). The results of luciferase, RNA immunoprecipitation, and RNA pull-down assays identified that LINC00221 interacted with let-7a-5p to increase expression of MMP11. Furthermore, we demonstrated that LINC00221 silencing increased let-7a-5p and inhibited MMP11 expression, thereby delaying the progression of HCC in vitro.

**Conclusions:**

Silencing of LINC00221 could prevent HCC progression via upregulating let-7a-5p and downregulating MMP11. As such, LINC00221 inhibition presents a promising antitumor strategy for the treatment of HCC.

## Background

Hepatocellular carcinoma (HCC) is the predominant histological subtype among primary liver cancers, and ranks 2nd among cancer-related deaths in China [1, 2]. HCC usually occurs in a condition of chronic inflammation [3]. Most cases of HCC arise in developing countries, but the global incidence is increasing in parallel with its risk factors, namely obesity, alcoholism, as well as hepatitis C [4]. The prospects for long-term survival with HCC remains poor [5], since most available treatment protocols do little to alter the poor prognosis, and only one third of cases are eligible for curative treatments [6]. Therefore, there is an urgent need to obtain a better understanding of the genetic and physiological factors that mediate the progression of HCC.

Multiple long non-coding RNAs (lncRNAs) possess the vital potential to control cellular process involved in the pathophysiology of HCC [7]. LncRNAs are a group of non-protein-coding transcripts with a length of 200 or more nucleotides; dysregulation of lncRNAs possibly contributes to development and progression of tumors [8]. Long intergenic noncoding RNA 00,221 (LINC00221), also known as NCRNA00221 [9], is an lncRNA recognized to be a potential prognostic biomarker for HCC [10]. However, its involvement in HCC progression remains to be established. LncRNAs have been widely identified to function as competitive endogenous RNAs (ceRNAs) or sponges of microRNAs (miRNAs), thus mediating effects on the post-transcription regulation of mRNAs [11]. For example, a previous study has suggested that the lncRNA Ras suppressor protein 1 pseudogene 2 (RSU1P2) mediates gene expression in cervical cancer by functioning as a ceRNA of let-7a [12]. Importantly, miRNA let-7a exhibits low expression levels in HCC cells [13]. Other studies show that silencing of lncRNA LINC01561 can suppress proliferation while enhancing the apoptosis of BrCa cells by increasing miR-145-5p and decreasing matrix metalloproteinase 11 (MMP11) expression [14]. MMP11 acts as a regulator of HCC proliferation and metastasis [15]. The results from the RNA22, Starbase, mirDIP, and miRDB databases, predicted that LINC00221 could bind to let-7a-5p, which in turn could bind to MMP11. Therefore, we hypothesized that LINC00221 might have a crucial modulatory effect on the growth of HCC cells through the let-7a-5p/MMP11 axis.

## Methods

### Microarray-based RNA expression profiling

We downloaded microarray profiles and annotation probe files related to HCC from the Gene Expression Omnibus (GEO) database (Table 1). The Affy package of R software was employed to perform background correction and normalization processing for the microarray data [16]. Using |log2 (fold change)|> 1 and value < 0.05 as threshold criteria, the Limma package (http://master.bioconductor.org/packages/release/bioc/html/limma.html) was applied to screen out the differentially expressed lncRNAs/miRNAs/mRNAs*.* HCC-related lncRNAs were screened from the GSE33006 microarray, which contained data from three pairs of HCC tissue samples and adjacent normal liver tissues. The downstream regulatory miRNAs of candidate lncRNA were predicted using the RNA22 database, which were intersected with the down-regulated miRNAs in HCC screened from GSE12717 to select candidate miRNAs. The target genes of candidate miRNAs were obtained from the Starbase, mirDIP, and miRDB databases, and then were intersected with up-regulated genes in HCC from the GSE89377 and GSE117361 microarrays using the Venn website. Data of HCC prognosis and HCC-related gene expression from The Cancer Genome Atlas (TCGA) database were further analyzed using R software. The package edgeR of R software was applied for differential analyses of the transcriptome profiling data [17], and false positive discovery (FDR) correction was utilized with the p-value defined by the package multi-test. The differentially expressed genes (DEGs) were screen out with the threshold of FDR < 0.05 and |log2 (fold change)|> 2.

**Table 1:** HCC-related microarray profiles

GSE	Sample size	Platforms
GSE33006	3 tumor tissue samples; 3 adjacent normal tissue samples	GPL570
GSE12717	10 tumor tissue samples; 6 adjacent normal tissue samples	GPL7274
GSE89377	35 tumor tissue samples; 13 adjacent normal tissue samples	GPL6947
GSE117361	2 tumor tissue samples; 2 adjacent normal tissue samples	GPL6480

### Study subjects

The HCC tissues as well as the adjacent non-tumor tissue (adjacent to the tumor margin at a distance greater than 2 cm) were collected from 45 HCC patients and subsequently stored in liquid nitrogen. The enrolled patients included 26 males and 19 females (aged 32–69 years), with 17 cases having a tumor size ≤ 5 cm and the remaining 28 cases with tumor size > 5 cm; 15 cases had multiple tumors and 30 cases had a single tumor. The clinical stage of all patients was determined based on the Edmondson-Steiner grade [18], with 21 cases regarded as grade I/II and 24 cases as grade III. Based on the Tumor-Node-Metastasis (TNM) staging classification of Modified Union for International Cancer Control (mUICC) stage [19], 28 cases were confirmed to be stage I/II and 17 cases as stage IIIa. The inclusion criteria were based on the following clinical manifestations: (1) alpha-fetoprotein (AFP) level maintained ≥ 200 µg/mL for one month; (2) AFP level maintained ≤ 200 µg/mL for two months [20]; (3) The diagnosis of HCC was confirmed by means of abdominal CT scans on a Philips Brilliance CT 64-channel scanner (Philips Medical Systems, Eindhoven, the Netherlands) [21]. The exclusion criteria were as follows: patients who had a history of chemotherapy or radiotherapy, or who had participated in other clinical trials (including placebo-controlled trials) within three months before the experiment, had received immunotherapy, or suffered from hepatitis B virus (HBV) or primary biliary cirrhosis [22].

### Fluorescence in situ hybridization (FISH) assay

A FISH assay was conducted to identify the subcellular localization of LINC00221 in the HCC cells [23]. HCC cells were seeded in a 24-well plate at a concentration of 5 × 10^3^ cells per well. After 24 h, the supernatant was removed, and the cells were fixed with 4% paraformaldehyde, permeated in 0.1% Triton X-100, and then treated with pre-hybridization solution at 37 °C. The double-stranded DNA probe was incubated in a water bath at 75 °C for 5 min and then immediately placed on ice for denaturation. 5–10 min later, the prepared chromosome slide specimen was denatured, and 10 µL denatured or pre-annealed biotin-labelled LINC00221 probe (Shanghai Gefan Biotechnology Co., Ltd., Shanghai, China) was dropped on the denatured and dehydrated slide specimen. The specimen was then covered with an 18 × 18 coverslip, sealed with parafilm, and was placed in a humidified cassette at 37 °C to hybridize overnight (approximately 15–17 h). The next day, elution was then carried out to remove non-specifically bound probes, followed by hybridization signal amplification. Ten minutes later, samples were analyzed under an Axio Observer A1 inverted microscope (Carl Zeiss, Jena, Germany).

### Cell transfection

The human normal liver cell line (HL-7702) and three HCC cell lines (MHCC97-H, Huh7, and Hep3B) obtained from Shanghai Institutes for Biological Sciences, Chinese Academy of Sciences (Shanghai, China) were employed for this study. The cells were incubated with Dulbecco's modified Eagle’s medium supplemented with 20% fetal bovine serum, penicillin (50 U/mL) and streptomycin (50 U/mL) at 37 °C with 5% CO_2_, followed by sub-culture until the cells attained a logarithmic growth rate. HL-7702, MHCC97-H, Huh7, and Hep3B cells were incubated in a 6-well plate until attaining 80% confluence. The cells at passage three were utilized for cell line selection. Expression patterns of LINC00221 were determined by reverse transcription-quantitative polymerase chain reaction (RT-qPCR). Next, the cell lines MHCC97-H and Huh7 with high LINC00221 expression were assigned into blank (cells without transfection), si-LINC00221 (cells transfected with siRNA against LINC00221), and si-negative control (NC) groups (cells transfected with scramble siRNA). Lipofectamine 2000 (Invitrogen Inc., Carlsbad, CA) was employed for cell transfection.

According to the sequence information for LINC00221 (NR_122034.1) in the Genebank, we designed three LINC00221 sequences. After successful construction of the recombinant plasmid and verification by sequencing, the plasmid was extracted, and the cell concentration determined prior to preservation at − 20 °C for later use. Cells (5 × 10^3^ cells/well) were allowed to incubate in a 6-well plate for 24 h and subsequently transfected using Lipofectamine 2000. After a 48-h transfection period, transfection efficiency was visualized under a microscope (CFM-500E/CFM-500Z, Changfang Optical Instruments Co., Ltd., Shanghai, China).

### RT-qPCR

Total RNA was extracted using an RNA extraction kit (Takara Biotechnology Ltd., Dalian, China) based on the Trizol method. The absorbance (A) at 260 nm and 280 nm was measured with a spectrophotometer (PHLES, China). The high quality of the RNA was confirmed by an A260/A280 ratio in the range 1.9–2.0. The RNA reverse transcription was conducted with the application of TaqMan microRNA assay RT primer (4427975, Applied Biosystems, Foster City, CA) and Primescript RT reagent kits (rr047a, Takara, Tokyo, Japan). The primers were designed and synthesized by Takara (Table 2). GAPDH was used as internal reference for LINC00221, while U6 was used for let-7a-5p. PCR was conducted on an ABI7500 quantitative PCR instrument (Applied Biosystems). The reaction conditions consisted of pre-denaturation at 95 °C for 10 min, 40 cycles of 10 s denaturation at 95 °C, 20 s annealing at 60 °C, and 34 s extension at 72 °C. The 2^−ΔΔCt^ method was applied to determine the relative expression of the target. ΔΔCt = ΔCt (experimental group) − ΔCt (control group); ΔCt = Ct (target gene) − Ct (internal control).

**Table 2:** Primer sequences for RT-qPCR

Target	Sequence
LINC00221	Forward: 5′-TATGTGGTACAGGGTTGGGC-3′
	Reverse: 5′-TGCAGAGCCAACTCTCCTTC-3′
GAPDH	Forward: 5′-GGAGCGAGATCCCTCCAAAAT-3′
	Reverse: 5′-GGCTGTTGTCATACTTCTCATGG-3′
let-7a-5p	Forward: 5′-UGAGGUAGUAGGUUGUAUAGUU-3′
	Reverse: 5′-CAGTGCGTGTCGTGGAGT-3′
U6	Forward: 5′-CTCGCTTCGGCAGCACA-3′
	Reverse: 5′-AACGCTTCACGAATTTGCGT-3′

*LINC00221* long intergenic noncoding RNA 00221, *GAPDH* glyceraldehyde-3-phosphate dehydrogenase, *RT-qPCR* reverse transcription-quantitative polymerase chain reaction

### Western blot analysis

The total protein extraction from tissues or cells was conducted with radioimmunoprecipitation assay (RIPA) lysis buffer (P0013c, Beyotime Biotechnology, Shanghai, China) containing phenyhnethylsulfonyl fluoride (PMSF). The supernatant was incubated on ice for 30 min, and centrifuged at 4 °C and 8000 *g* for 10 min, followed by bicinchoninic acid (BCA) protein assay (CWBio, Beijing, China). The extracted proteins were stored at – 80 ℃ after packaging. The proteins were separated by sodium dodecyl sulfate–polyacrylamide gel electrophoresis (SDS-PAGE) for 1 h, transferred onto nitrocellulose membranes, and then blocked with 5% skimmed milk (PBS) for 2 h. The membranes were incubated for 30 min at ambient temperature with rabbit polyclonal antibodies MMP11 (1:1000, ab119284), Bcl-2 (1:1000, ab182858), Cyclin D1 (1:1000, ab40754), CDK4 (1:1000, ab199728), Bax (1:1000, ab182733), and GAPDH (ab9485, 1:2500) (Abcam, Cambridge, UK). HRP-coupled antibody to immunoglobulin G (IgG) (A21020, 1:5000, AmyJet Scientific Inc., Wuhan, China) was loaded for 1-h incubation. The immunoblots were subsequently developed using enhanced chemiluminescence reagent (BB-3501, Amersham Biosciences, Piscataway, NJ), and imaged on the gel imager. An imaging system (Bio-Rad Inc., Hercules, CA) was utilized for gray value analysis with the application of Quantity One v4.6.2 software [24].

### 3-(4,5-dimethylthiazol-2-Yl)-2,5-diphenyltetrazolium bromide (MTT) assay

After the transfection process, the cells were trypsinized. The cell suspension (5 × 10^3^ cells/well) in a 96-well plate was added with 20 μL MTT solution (GD-Y1317, Guduo Biotechnology Company, Shanghai, China) per well at 24, 48 and 72 h post transfection, respectively. The supernatant was aspirated after centrifugation, followed by the addition of 150 μL dimethylsulfoxide (DMSO; D5879-100ML, Sigma-Aldrich, SF, CA) into each well. The optical density (OD) value was measured at 490 nm using a microplate reader (DG5031, Shanghai Bajiu Industrial Co., Ltd., Shanghai, China) [25]. Cell viability = (OD value_experimental group_ − OD_blank group_) / (OD_control group_ − OD_blank group_) × 100%.

### Flow cytometry

After transfection for 48 h, the cells were fixed, centrifuged, and dyed with 500 μL solution containing propidium iodide (PI, 50 μg/mL) (88378, Sigma-Aldrich) and RNase A (100 μg/mL) (GE101-01, TransGen Biotech Co., Ltd., Beijing, China). A CytoFLEX flow cytometer (excitation wavelength: 488 nm) was utilized to analyze the cell cycle.

Forty-eight h post transfection, the prepared Annexin-V/PI dying solution with Annexin-V-fluorescein isothiocyanate (FITC), propidium iodide (PI) and N-2-hydroxyethylpiperazine-N′-2-ethanesulfonic acid (HEPES) buffer (1:2:50) were incubated with cells together for 10 min. The cells were probed with fluorescent solution SA-FLOUS (Origin Biosciences Inc., Nanjing, China) after centrifugation. To assess cell apoptosis, the fluorescence was detected using a 620-nm bandpass filter for PI and a 525-nm bandpass filter for FITC at 488-nm excitation wavelength, respectively [26].

### Scratch test

Uniform horizontal lines were drawn on the surface of the 6-well plates. Scratches perpendicular to the horizontal lines were subsequently made on the cell monolayers. Images were obtained at 0 and 24 h of incubation using an inverted microscope, and the cell migration distance between the scratches was measured accordingly [27].

### Transwell assay

Transwell assay was conducted in a Transwell chamber by conventional means, with staining with crystal violet for 20 min, whereupon the cells that had migrated through the Matrigel were counted in three randomly selecting fields from each sample [28].

### RNA immunoprecipitation (RIP)

The binding between LINC00221 and Argonaute 2 (Ago2) protein as well as that between let-7a-5p and Ago2 was assessed using a RIP kit (Millipore Company, Billerica, MA). The cells were lysed by incubation in RIPA lysis buffer (P0013B, Beyotime Biotechnology) in an ice bath, followed by 10-min centrifugation at 14,000 rpm at 4 ℃. For each coprecipitation reaction system, 50 μL magnetic beads were resuspended with 100 μL RIP wash buffer, with addition of 5 μg antibody: rabbit anti-Ago2 (1:100, ab32381, Abcam) or rabbit anti-human IgG (1:100, ab109489, Abcam) (used as NC). The obtained bead-antibody complex was then incubated overnight at 4 °C with cell lysate (100 μL). One portion of the cell lysate was employed as the input. The samples and input were detached by protease K to extract RNA, followed by RT-qPCR.

### RNA pull-down

Transfection was performed with the wild type (WT)-biotinylated LINC00221 and mutant type (MUT)-biotinylated LINC00221 (50 nM for each) for 48 h. Next, the cell lysate was reacted at 4 °C with M-280 streptavidin magnetic beads (Sigma-Aldrich) that was precoated with RNase-free yeast tRNA (Sigma-Aldrich). 3 h later, the beads were rinsed. Finally, the expression of let-7a-5p in the eluted RNA was determined by RT-qPCR.

### Dual-luciferase reporter assay

The cells (2 × 10^5^ cells/well) in 6-well plates were transfected, and cultured for 48 h. The luciferase activities of LINC00221 and MMP11 in the cells were determined through application of the Genecopoeia dual-luciferase detection kit (D0010, Solarbio, Beijing, China). The luminance was detected using the Glomax20/20 luminometer provided by Promega (E5311, Zhongmei Biotechnology Co., Ltd., Shanxi, China).

### Animal treatment

Forty-eight healthy nude mice (aged 6–8 weeks, weighing about 18–22 g) were purchased from the Department of Pharmacology, Institute of Materia Medica, Chinese Academy of Medical Sciences (Beijing, China). Mice were separately caged for 7 days in a specific-pathogen-free animal laboratory with 60–65% humidity, 22–25 °C, and free access to feed and water, under a 12 h light/dark cycle. Mice were subcutaneously inoculated with 1 × 10^6^ cells/µL of cell suspension. Specifically, Huh7 and MHCC97-H cells (2 × 10^5^ cells/well) were seeded into a 6-well cell culture plate. Upon attaining 30% confluence, cells were infected with lentivirus (2 × 10^6^ TU) expressing short hairpin RNA (sh)-NC, sh-LINC0022, oe-NC, or oe-LINC0022. The lentivirus was mixed with 5 μg poly-brene, which was added into 1 mL serum-free and anti-bacterial medium. The infection efficiency was monitored under an inverted fluorescence microscope for 2–3 days. After 48 h of infection, puromycin (1 μg/mL) was added into each well to select stably infected cells. The cells were further cultured with complete medium after obtaining the stably infected cells. The tumor volume of each mouse was measured every seven days for five weeks, whereupon the mice were euthanized, and the tumor was removed and weighted.

### Statistical analysis

SPSS 21.0 software (IBM Corp., Armonk, NY) was utilized for data analysis (*p* < 0.05 was defined as statistically significant). Measurement data were expressed as mean ± standard deviation. All experiments were independently conducted three times, and all data were evaluated for normality and homogeneity of variance. Comparison between cancerous and adjacent non-cancerous tissues was made using paired *t-*test while that between other two groups was performed by unpaired *t*-test. Multiple groups were compared by one-way analysis of variance (ANOVA) with Tukey’s post hoc test. The repeated measures ANOVA was performed for the comparison of tumor volume at different time points, whereas two-way ANOVA was conducted for comparing cell viability at indicated time points, followed by Bonferroni post hoc test.

## Results

### LINC00221 is highly expressed in HCC

**Fig. 1:**
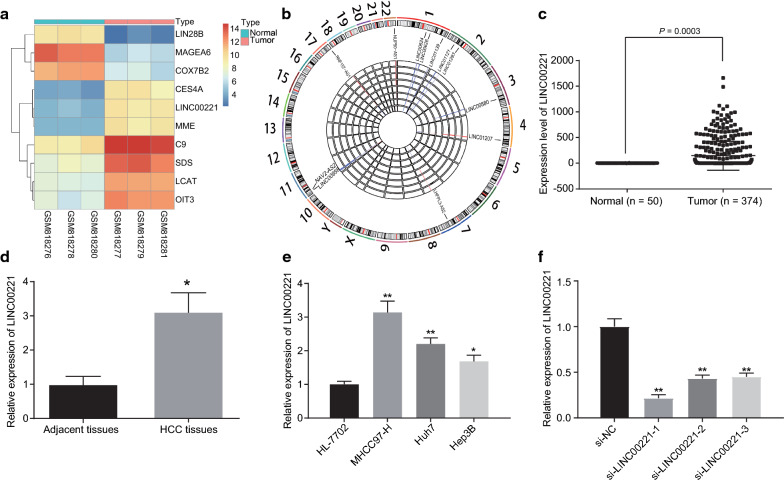
LINC00221 is robustly induced in HCC. **a** heat maps with hierarchical clustering analysis for microarray GSE33006 obtained from GEO database (http://www.ncbi.nlm.nih.gov/geo). The abscissa indicates different individuals, the ordinate on the right indicates the gene; the left dendrogram refers to clustering. The upper figure shows whether the sample type consists of normal or tumor; the histogram in the upper right refers to color gradation, which indicates gene expression. The color refers to the analysis of DEGs expression, with each square representing the expression of one gene in one sample. **b** Circos map of microarray GSE33006. **c** LINC00221 expression in HCC from TCGA database (http://cancergenome.nih.gov/). **d** LINC00221 expression in the HCC tissues and adjacent non-tumor tissues examined by RT-qPCR (n = 45). **e** the expression of LINC00221 in 3 MHCC97-H, Huh7 and Hep3B cell lines determined by RT-qPCR. **f** Silencing efficiency of LINC00221 examined by RT-qPCR. Cell experiments were independently repeated 3 times. *p < 0.05; **p < 0.05. Comparison between two groups was made by paired *t-*test while comparison among multiple groups was made using one-way ANOVA with Tukey’s post hoc test

Microarray analysis (GSE33006) revealed an upregulation of LINC00221 in HCC (Fig. 1a–c). Analysis by RT-qPCR demonstrated noticeably higher expression of LINC00221 in the HCC tissues versus adjacent non-tumor tissues (Fig. 1d). LINC00221 expression in three HCC cell lines (MHCC97-H, Huh7, and Hep3B) was measured by RT-qPCR. Consistently, higher LINC00221 expression was observed in Huh7 and MHCC97-H cell lines than in HL-7702, so these two cell lines were selected for subsequent cell experiments (Fig. 1e). After cell transfection, si-LINC00221-1, si-LINC00221-2 and si-LINC00221-3 decreased the LINC00221 expression, among which the si-LINC00221-1 treatment exhibited the best silencing efficiency (Fig. 1f). Therefore, the si-LINC00221-1 was selected for the LINC00221 loss-of-function experiments.

After correlation analysis, we determined that LINC00221 expression was highly correlated to lymph node metastasis (LNM), clinical grade, TNM staging, and AFP content (Table 3). Patients at stage IIIa HCC exhibited a lower positive expression of LINC00221 (37.78%) than that those at stage I/II HCC (62.22%). The positive expression of LINC00221 in grade I//II patients (46.67%) was lower than that in patients at grade III (53.33%). Positive expression of LINC00221 in patients with LNM (24.44%) was lower than that in patients without LNM (75.56%), while the positive expression of LINC00221 in patients with an AFP greater than 200 ng/mL (53.33%) was higher than those with an AFP less than or equal to 200 ng/mL (46.67%). However, LINC00221 expression displayed no marked difference in terms of gender, age, or tumor volume and number.

**Table 3:** The correlation of LINC00221 expression with the progression of HCC

Clinicopathological characteristics	Case	LINC00221 expression	*p*
Age (years)			0.689
≤ 45	16	3.076 ± 0.714	
> 45	29	3.148 ± 0.512	
Gender			0.824
Male	26	3.139 ± 0.540	
Female	19	3.099 ± 0.654	
Tumor size			0.443
≤ 5 cm	17	3.035 ± 0.631	
> 5 cm	28	3.175 ± 0.560	
Tumor number			0.282
Single	30	3.055 ± 0.522	
Multiple	15	3.256 ± 0.693	
Clinical grade			0.001
I/II	21	2.822 ± 0.704	
III	24	3.385 ± 0.262	
TNM stage			0.005
I/II	28	2.936 ± 0.639	
III	17	3.429 ± 0.300	
LNM			0.001
Yes	11	3.517 ± 0.593	
No	34	2.994 ± 0.336	
AFP (ng/mL)			< 0.001
≤ 200	21	2.824 ± 0.706	
> 200	24	3.383 ± 0.262	

*HCC* hepatocellular carcinoma, *TNM* tumor node metastasis, *LNM* lymph node metastasis, *AFP* alpha-fetoprotein

### Repression of LINC00221 inhibits proliferation and enhances apoptosis of HCC Cells

**Fig. 2:**
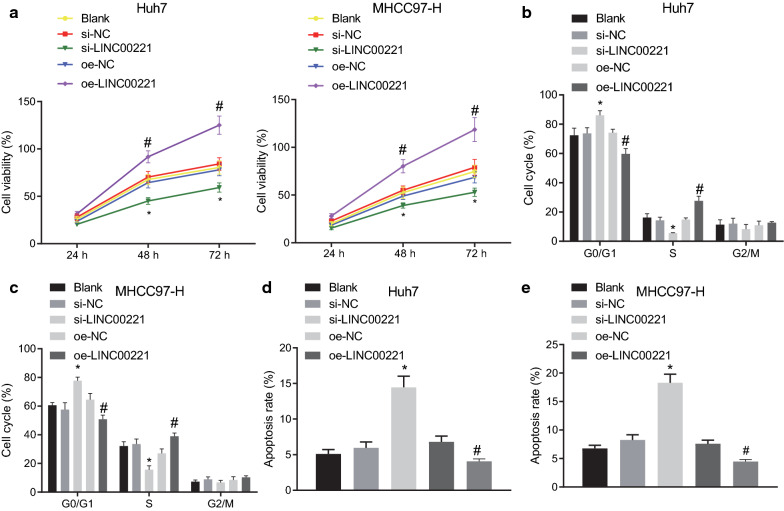
Suppression of LINC00221 inhibits cell viability and cell cycle entry while enhancing cell apoptosis in HCC (Huh7 and MHCC97-H) cells. **a** viability of Huh7 and MHCC97-H cells upon LINC00221 silencing or overexpression assessed by MTT assay. B-C, cell cycle distribution in Huh7 (**b**) and MHCC97-H (**c**) cells upon LINC00221 silencing or overexpression examined by flow cytometry. D-E, the apoptosis in Huh7 (**d**) and MHCC97-H (**e**) cells after silencing or overexpression of LINC00221 examined by flow cytometry. Cell experiments were independently repeated 3 times. *p < 0.05 *vs.* the si-NC group; ^#^*p* < 0.05 *vs.* the oe-NC group. Comparison among multiple groups was made using one-way ANOVA with Tukey’s post hoc test while data at different time points were compared using two-way ANOVA with Bonferroni post hoc test

As depicted in Fig. 2a, the results of the MTT assay revealed no significant difference at the 24^th^ h post transfection between the Huh7 and MHCC97-H cells. Cell viability was notably lowered by si-LINC00221 but enhanced by oe-LINC00221 in both cell lines. Additionally, as revealed by the PI single staining results (Fig. 2b, c, Additional file 1: Figure S1A, B), there was no significant difference concerning cell cycle in either cell line among blank, si-NC and oe-NC groups. si-LINC00221 treatment resulted in an elevated proportion of the cells at the G1 phase, but a reduced proportion of the cells at the S phase, while oe-LINC00221 led to enhanced cell cycle entry both in Huh7 and MHCC97-H cells. Annexin V/PI double staining results displayed no significant difference among the blank, si-NC and oe-NC groups in either cell line. However, their apoptosis rates were elevated by si-LINC00221 but reduced by oe-LINC00221 treatment (Fig. 2d, e, Additional file 1: Figure S1C, D). Therefore, silencing LINC00221 could inhibit cancer progression in HCC cells.

### Repression of LINC00221 reduces the migration and invasion of HCC cells

**Fig. 3:**
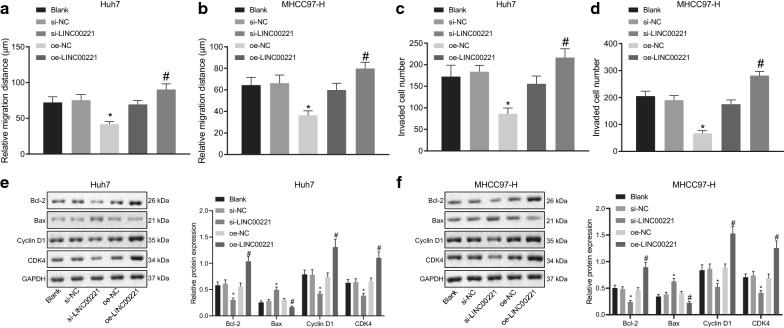
Downregulation of LINC00221 restrains migration and invasion of HCC (Huh7 and MHCC97-H) cells. **a**, **b** migration distance of Huh7 (**a**) and MHCC97-H (**b**) cells assessed by scratch test. **c**, **d** number of invaded Huh7 (**c**) and MHCC97-H (**d**) cells examined by Transwell assay. **e**, **f** Bcl-2, Bax, Cyclin D1, and CDK4 protein expression in Huh7 (**e**) and MHCC97-H (**f**) cells measured by Western blot analysis. Cell experiments were independently repeated 3 times. *p < 0.05 *vs.* the si-NC group; ^#^*p* < 0.05 *vs.* the oe-NC group. Comparison among multiple groups was made using one-way ANOVA with Tukey’s post hoc test

As revealed by the data from Scratch test and Transwell assay, the Huh7 and MHCC97-H cells exhibited consistent changes following alternations of their LINC00221 expression. As illustrated in Fig. 3a, b, Additional file 2: Figure S2A, B, Fig. 3c, d, and Additional file 2: Figure S2C, D, si-LINC00221 induced significantly decreased cell migration distance and fewer invasive cells, while oe-LINC00221 caused enhanced cell migration and invasion.

The expression patterns of anti-apoptotic protein Bcl-2, pro-apoptotic protein Bax, and cell cycle markers cyclin D1 and CDK4 in Huh7 cells and MHCC97-H cells were measured by immunoblotting. The expression of Bcl-2, cyclin D1 and CDK4 was significantly decreased, while that of Bax was increased upon si-LINC00221 treatment. However, the expression of Bcl-2, cyclin D1 and CDK4 was enhanced, while Bax expression was reduced in response to oe-LINC00221 treatment (Fig. 3e, f). Taken together, the above findings led us to conclude that silencing LINC00221 suppressed the migrating and invading properties of HCC cells.

### Inhibition of LINC00221 suppresses tumor growth in HCC

**Fig. 4:**
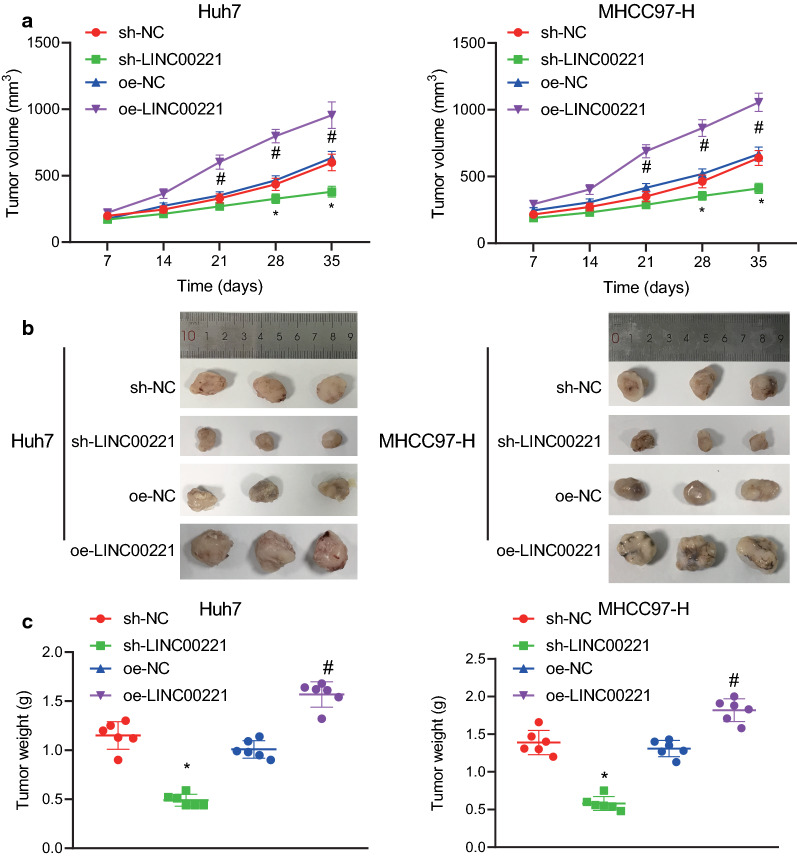
Suppression of LINC00221 inhibits tumor growth in vivo. **a** Tumor volume in nude mice; **b** Representatives images of transplanted tumors formed in nude mice; **c** Tumor weight. N = 6. *p < 0.05 *vs.* the sh-NC group; ^#^*p* < 0.05 *vs.* the oe-NC group. Comparison among multiple groups was made using one-way ANOVA with Tukey’s post hoc test while data at different time points were compared using repeated measures ANOVA with Bonferroni post hoc test

We further investigated whether LINC00221 could function in vivo. LINC00221-deficient or LINC00221-overexpressed Huh7/MHCC97-H cells were transplanted into HCC nude mice. The expression of LINC00221 was markedly reduced in the tumor xenografts of nude mice transplanted with LINC00221-deficient Huh7/MHCC97-H cells, while its expression was considerably elevated in the tumor xenografts of mice transplanted with Huh7/MHCC97-H cells overexpressing LINC00221 (Additional file 3: Figure S3). The volume and weight of transplanted tumors were significantly reduced by LINC00221 knockdown, yet the volume and weight of transplanted tumors were notably elevated upon enhanced LINC00221 expression (Fig. 4a–c). These findings suggested that silencing of LINC00221 was capable of suppressing tumor growth in vivo*.*

### LINC00221 upregulates the expression of MMP11 by competitively binding to Let-7a-5p

**Fig. 5:**
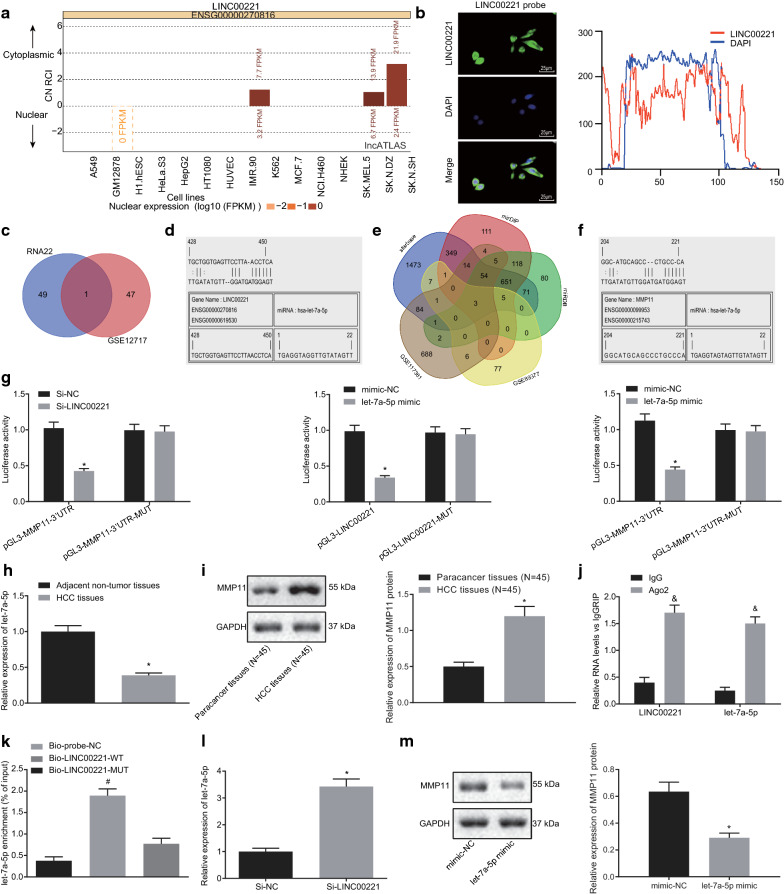
LINC00221 upregulates the MMP11 expression by competitively binding to let-7a-5p. **a** LncATLAS website for lncRNA subcellular localization revealed that LINC00221 was mainly located in the cytoplasm. **b** The subcellular localization of LINC00221 verified by FISH assay (× 400; the left) and the co-localization profile intensity (the right). **c** Venn map (http://bioinformatics.psb.ugent.be/webtools/Venn/) showing downstream regulatory miRNAs of LINC00221 predicted by RNA22 database (https://cm.jefferson.edu/rna22/Precomputed/) (blue circle) and downregulated miRNAs in HCC samples from GSE12717 microarray (red circle). **d** The binding site between LINC00221 and let-7a-5p sequences predicted on the RNA22 website. **e** Venn map showing downstream target genes of let-7a-5p predicted by Starbase (http://starbase.sysu.edu.cn/index.php) (blue circle), mirDIP (http://ophid.utoronto.ca/mirDIP/) (red circle), and miRDB (http://www.mirdb.org/) (green circle), and upregulated genes in HCC samples from GSE89377 microarray (yellow circle) and from GSE117361 microarray (orange circle). **f** The binding site between let-7a-5p and MMP11 predicted on the RNA22 website. **g** The relationships among LINC00221, let-7a-5p and MMP11 identified by dual-luciferase reporter gene assay. **h** The let-7a-5p expression in HCC and adjacent non-tumor tissues (n = 45) examined by RT-qPCR. **i** The MMP11 protein expression in HCC and adjacent non-tumor tissues (n = 45) measured by Western blot analysis. **j** The relationships among LINC00221, let-7a-5p and Ago2 determined by RIP assay. **k** The binding of LINC00221 to let-7a-5p identified by RNA pull-down. **l** The regulatory effect of LINC00221 on let-7a-5p expression examined by RT-qPCR. **m** MMP11 protein expression after upregulation of let-7a-5p measured by western blot analysis. Cell experiments were independently repeated 3 times. *p < 0.05 *vs.* the mimic NC group, si-NC group or adjacent non-tumor tissues (n = 45); ^&^*p* < 0.05 *vs.* the IgG group; ^#^*p* < 0.05 *vs.* the Bio-LINC00221-MUT group. Comparison between HCC and adjacent non-tumor tissues was made using paired *t-*test while comparison between other two groups was performed by unpaired *t*-test

Analysis of an lncRNA subcellular localization website (http://lncatlas.crg.eu/) [29] revealed that LINC00221 was predominately located in the cytoplasm (Fig. 5a). Through the application of FISH, as shown in Fig. 5b, the green-stained LINC00221 probe was mainly located in the cytoplasm, while the nucleus was stained in blue. In merged images, green fluorescence was clearly observed in the cytoplasm, indicative of the localization of LINC00221 in the cytoplasm.

The downstream regulatory miRNAs predicted by the RNA22 database were intersected with the downregulated miRNAs in HCC samples from the GSE12717 microarray. Only let-7a-5p was present at the intersection (Fig. 5c). We also observed a specific binding site between LINC00221 and let-7a-5p sequences (Fig. 5d). To explore further the downstream mechanism of this interaction, we employed Starbase, mirDIP and miRDB databases to predict the targets of let-7a-5p. By intersecting those target genes with upregulated genes in HCC samples from GSE89377 and GSE117361 microarrays, we identified MMP11, COL1A1 and COL1A2 (Fig. 5e), among which MMP11 has rarely been reported in previous studies on HCC. Moreover, we found a specific binding site between let-7a-5p and the MMP11 sequence (Fig. 5f). The above results suggested that LINC00221 might regulate MMP11 expression via binding to let-7a-5p. To validate this, we then applied a luciferase assay. Results of this assay (Fig. 5e) demonstrated that the luciferase activity of pGL3-LINC00221 or pGL3-MMP11-3′UTR was decreased by co-transfection with let-7a-5p mimic, while that of pGL3-MMP11-3′UTR was also decreased after co-transfection with si-LINC00221. Then, we found that the HCC tissues displayed markedly decreased let-7a-5p expression levels accompanied by elevated MMP11 protein expression relative to adjacent non-tumor tissues (Fig. 5f, g). The application of a RIP assay revealed that, in contrast to the IgG group, the Ago2 group had significantly higher levels of LINC00221 and let-7a-5p expression (Fig. 5h). The application of an RNA pull-down exhibited that, compared with the Bio-LINC00221-MUT group, the Bio-LINC00221-WT group exhibited significantly increased let-7a-5p expression (Fig. 5i). After inhibition of LINC00221, the level of let-7a-5p expression markedly decreased (Fig. 5j), while MMP11 protein expression was conspicuously decreased upon let-7a-5p mimic transfection (*p* < 0.05) (Fig. 5k). Taken then above, we concluded that LINC00221 upregulated the MMP11 expression by competitively binding to let-7a-5p.

### Knock-down of LINC00221 represses the aggressiveness but enhances apoptosis of HCC cells by inhibiting MMP11 via upregulation of Let-7a-5p

**Fig. 6:**
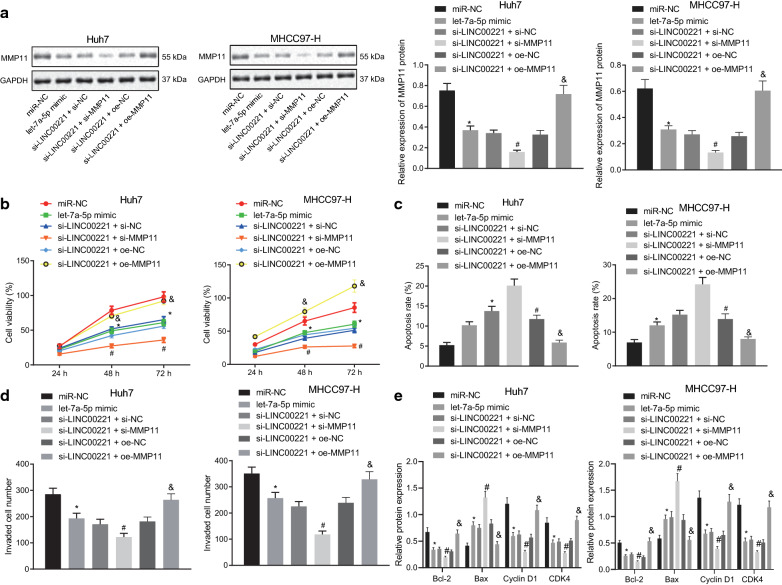
Silencing of LINC00221 inhibits the proliferation and invasion but accelerates apoptosis of HCC (Huh7 and MHCC97-H) cells by upregulating let-7a-5p and suppression of MMP11. The Huh7 and MHCC97-H cells were transfected with let-7a-5p mimic, si-LINC00221 or co-transfected with si-LINC00221 and si-MMP11 or si-LINC00221 and oe-MMP11. **a** The MMP11 protein expression in Huh7 and MHCC97-H cells examined by Western blot analysis. **b** The proliferation of Huh7 and MHCC97-H cells assessed by MTT assay. **c** The apoptosis of Huh7 and MHCC97-H cells examined by flow cytometry. **d** Number of invaded Huh7 and MHCC97-H cells tested by Transwell assay. **e** Bcl-2, Bax, cyclin D1, and CDK4 protein expression in Huh7 and MHCC97-H cells measured by Western blot analysis. Cell experiment was independently repeated 3 times. *p < 0.05 *vs.* the miR-NC group; ^#^*p* < 0.05 *vs.* the si-LINC00221 + si-NC group; ^&^*p* < 0.05 *vs.* the si-LINC00221 + oe-NC group. Comparison among multiple groups was made using one-way ANOVA with Tukey’s post hoc test while data at different time points were compared using two-way ANOVA with Bonferroni post hoc test

Huh7 cells and MHCC97-H cells were transfected with let-7a-5p mimic to explore its effect on the progression of HCC. In particular, we conducted transfection with si-LINC00221 + si-MMP11 or with si-LINC00221 + oe-MMP11 to analyze whether LINC00221 participated in the growth of HCC by regulating the let-7a -5p/MMP11 axis. Results showed that let-7a-5p mimic treatment obviously decreased the MMP11 protein expression. MMP11 protein expression was appreciably lowered by si-MMP11, but rescued by oe-MMP11 treatment in the LINC00221-deficient cells (Fig. 6a). The MTT assay results as displayed in Fig. 6b revealed that cell proliferation was significantly diminished by let-7a-5p mimic, while a similar decrease was induced by si-MMP11 in the LINC00221-deficient cells. However, cell proliferation suppressed by si-LINC00221 was rescued by oe-MMP11. The flow cytometric data in Fig. 6c displayed that let-7a-5p mimic provoked significantly higher cell apoptosis, and that si-MMP11 induced an enhancement of cell apoptosis in LINC00221-deficient cells. On the other hand, cell apoptosis induced by si-LINC00221 was decreased by oe-MMP11. Furthermore, the Transwell assay revealed that cell invasion was markedly decreased by the let-7a-5p mimic treatment, with a similar reduction identified in the LINC00221-deficient cells upon MMP11 knockdown. Moreover, cell invasion suppressed by si-LINC00221 was enhanced by oe-MMP11 (Fig. 6d).

At the molecular level, the expression of Bcl-2, Cyclin D1 and CDK4 proteins was decreased, but Bax protein expression was upregulated by let-7a-5p mimic. In contrast to the si-LINC00221 + si-NC group, we observed reduced expression of Bcl-2, cyclin D1 and CDK4 proteins in the si-LINC00221 + si-MMP11 group, accompanied with elevated expression of Bax protein. Conversely, oe-MMP11 could partially reverse the changes in expression of the aforementioned proteins caused by si-LINC00221 (Fig. 6e).

The Huh7 and MHCC97-H cells exhibited similar changes regarding the aforementioned cellular behaviors and molecular levels. Taken together, the results indicated that LINC00221 silencing inhibited MMP11 by upregulating let-7a-5p, thus limiting the malignancy of HCC cells.

## Discussion

**Fig. 7:**
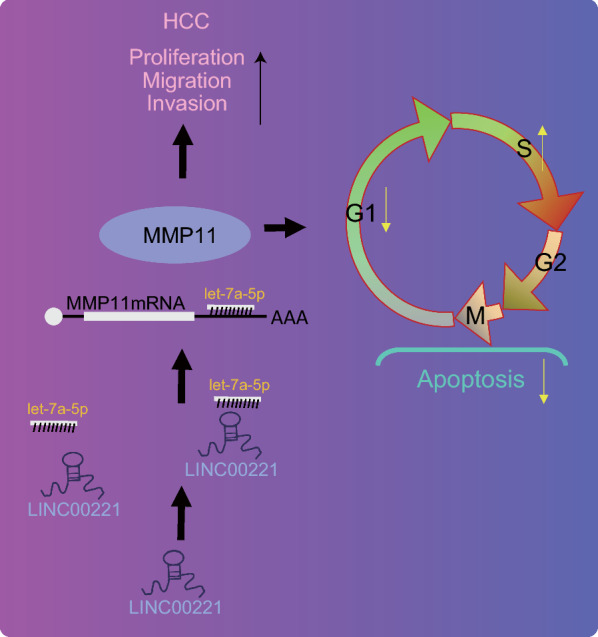
The mechanism involved LINC00221 in regulating the progression of HCC. LINC00221 was upregulated while let-7a-5p was downregulated in HCC cells, and highly expressed LINC00221 upregulated MMP11 expression by competitively binding to let-7a-5p, thereby promoting HCC cell viability, migration, and restraining cell apoptosis

In the progression of HCC, various genetic and epigenetic alterations regulate the function of proteins post-translationally, which in turn facilitate invasion and metastasis [30]. The present study elucidated the mechanisms associated with the regulatory effects of LINC00221 on HCC cellular behaviors. Our results suggested that LINC00221 promoted the progression of HCC via a mediation of the let-7a-5p/MMP11 axis (Fig. 7).

Aberrant expressions of lncRNAs are known for their contribution to hepatocarcinogenesis [31]. Our initial findings in this study revealed that LINC00221 was aberrantly increased in HCC tissues, and that this aberrant increase correlated with advanced LNM, clinical grade and TNM stage of HCC. However, LINC00221 silencing could impede the proliferative, invasive and oncogenic potentials of HCC cells and enhance their apoptosis. It is well-known that lncRNAs can sometimes act as ceRNAs, thus modulating the expression of different miRNAs in a cell-type dependent manner [32]. LINC00221, which is also involved in the ceRNA network in HCC [10], was further observed in this study to act as a ceRNA of let-7a-5p. Existing literature has indicated that let-7a has low expression in HCC cells [33], whereas let-7a overexpression suppresses growth, invasion, and sphere formation capability of HCC stem-like cells [34]. These literature results are partially consistent with our present findings that upregulating let-7a-5p could enhance malignant functions of HCC cells. Similarly, let-7a-5p expression is downregulated in lung cancer, and its decreased expression is conductive to the progression of this cancer [35].

Furthermore, the binding of miRNAs to lncRNAs decreased miRNA levels and led to increased expression of miRNA target genes [36]. The new evidence provided by our study also supported the notion that LINC00221 overexpression resulted in increased MMP11 levels by competitively binding to let-7a-5p. Previous data have suggested that LINC00221 sponges miR-519a to mediate the expression of ZBTB5, which is a mechanism involved in the regulation of cisplatin resistance in non-small-cell lung cancer (NSCLC) [37]. Another let-7 family member, let-7c, targets MMP11 to suppress metastasis in colorectal cancer [38]. Liu et al*.* have demonstrated that lncRNA nuclear paraspeckle assembly transcript 1 (NEAT1) negatively interacts with let-7a-5p to increase the expression of the let-7a-5p target gene Rsf-1 in NPC [39]. MMP11 has been proposed to be a hub cancer driver gene in lung adenocarcinoma [40]. Our present findings demonstrated that MMP11 exerted tumor-promotive functions on the progression of HCC. Indeed, a large number of previous studies have shown that MMP11 functions as an oncogene in a variety of cancers. For example, inhibition of MMP11 can impede the metastasis of gastric cancer [41]. Likewise, a previous study has shown that knockdown of MMP11 can hamper cancer progression in lung adenocarcinoma cells as well as impeding tumor growth in xenograft models [42]. More importantly, genetic variations in MMP11 may function as a reliable biomarker for the progression of HCC [43], while another study has reported that MMP11 contributes to HCC proliferation and metastasis by acting as the target gene of miR-125a [18]. In this study, we demonstrated that LINC00221 silencing suppressed the in vitro progression of HCC via upregulation of let-7a-5p and inhibition of MMP11 expression. Similarly, suppression of LINC01561 can inhibit cancer cell proliferation and facilitate apoptosis in breast cancer via increasing expression of miR-145-5p as well as reducing expression of MMP11 [17]. Additionally, overexpressing of lncRNA LOXL1-AS1 can accelerate cell proliferating and migrating processes, but restrain apoptosis via upregulating EGFR expression though inhibition of let-7a-5p [44]. Wang et al*.* have demonstrated that lncRNA antisense non-coding RNA in the INK4 locus (ANRIL) knockdown inhibits the tumorigenicity of NPC cells by modulating let-7a [45].

## Conclusion

We have elucidated the potential role of LINC00221 in HCC and provided new evidence that the silencing of LINC00221 contributed to prevention of HCC progression via let-7a-5p-targeted inhibition of MMP11. The demonstration of this mechanistic axis may provide novel insights for developing new targets for the improved treatment of HCC. Present results are encouraging for further investigations with larger sample size and with consideration of the confounding effects of targeted therapy with chemotherapy or chemoresistance on HCC.

## Supplementary Information

**Additional file 1: Figure S1.** Suppression of LINC00221 inhibits cell cycle entry while enhancing cell apoptosis in Huh7 and MHCC97-H cells. A-B, Representative images showing cell cycle in Huh7 (A) and MHCC97-H (B) cells upon LINC00221 silencing or overexpression examined by flow cytometry. C-D, Representative flow cytometric dot plots apoptosis in Huh7 (C) and MHCC97-H (D) cells after silencing or overexpression of LINC00221.

**Additional file 2: Figure S2.** Downregulation of LINC00221 restrains migration and invasion of HCC (Huh7 and MHCC97-H) cells. A-B, Representative images of migration distance of Huh7 (A) and MHCC97-H (B) cells assessed by scratch test. C-D, Representative images of invaded Huh7 (C) and MHCC97-H (D) cells examined by Transwell assay.

**Additional file 3: Figure S3.** Expression of LINC00221 in the tumor xenografts of nude mice transplanted with Huh7/MHCC97-H cells.

## Data Availability

The datasets generated/analyzed during the current study are available.

## Abbreviations

HCC: Hepatocellular carcinoma
lncRNAs: Long non-coding RNAs
TCGA: The Cancer Genome Atlas
GEO: Gene Expression Omnibus
ceRNA: Competitive endogenous RNA
miRNAs: MicroRNAs
RSU1P2: Ras suppressor protein 1 pseudogene 2
MMP11: Matrix metalloproteinase 11
FDR: False positive discovery
DEGs: Differentially expressed genes
TNM: Tumor-Node-Metastasis
mUICC: Modified Union for International Cancer Control
AFP: Alpha-fetoprotein
FISH: Fluorescence in situ hybridization
NC: Negative control
RT-qPCR: Reverse transcription quantitative polymerase chain reaction
GAPDH: Glyceraldehyde-3-phosphate dehydrogenase
OD: Optical density
RIP: RNA Immunoprecipitation
WT: Wild type
MUT: Mutant type
LNM: Lymph node metastasis
NSCLC: Non-small-cell lung cancer
NEAT1: Nuclear paraspeckle assembly transcript 1
